# Field-emission electron gun for a MEMS electron microscope

**DOI:** 10.1038/s41378-021-00268-9

**Published:** 2021-06-01

**Authors:** Michał Krysztof

**Affiliations:** Department of Microsystems, Faculty of Microsystem Electronics and Photonics, Wroclaw University of Science and Technology, ul. Z. Janiszewskiego 11/17, 50-372 Wroclaw, Poland

**Keywords:** Electronic devices, Sensors

## Abstract

This article presents a field-emission electron gun intended for use in a MEMS (microelectromechanical system) electron microscope. Its fabrication process follows the technology of a miniature device under development built from silicon electrodes and glass spacers. The electron gun contains a silicon cathode with a single very sharp protrusion and a bundle of disordered CNTs deposited on its end (called a sharp silicon/CNT cathode). It was tested in diode and triode configurations. For the diode configuration, a low threshold voltage <1000 V and a high emission current that reached 90 µA were obtained. After 30 min of operation at 900 V, the emission current decreased to 1.6 µA and was stable for at least 40 min, with RMS fluctuation in the anode current lower than 10%. The electron beam spot of the source was observed on the phosphor screen. In the diode configuration, the spot size was the same as the emission area (~10 µm), which is a satisfactory result. In the triode configuration, an extraction electrode (gate) control function was reported. The gate limited the emission current and elongated the lifetime of the gun when the current limit was set. Moreover, the electron beam current fluctuations at the anode could be reduced to ~1% by using a feedback loop circuit that controls the gate voltage, regulating the anode current. The developed sharp silicon/CNT cathodes were used to test the MEMS electron source demonstrator, a key component of the MEMS electron microscope, operating under atmospheric pressure conditions. Cathodoluminescence of the phosphor layer (ZnS:Ag) deposited on the thin silicon nitride membrane (anode) was observed.

## Introduction

The electron beam parameters of an electron microscope, i.e., a very small spot size with high beam density and stability, determine the quality of the image obtained. These parameters depend on the electron gun. The choice of the electron gun is especially important in the realization of miniature electron-optical columns (microcolumns). Field cathodes are best suited for the fabrication of microcolumns due to their high brightness and small initial source size^1^. For SEM applications, RMS fluctuations of a few percent over 30 min at a relatively low angular emission current density of <10 µA/sr are generally considered acceptable^1^. Field-emission cathodes are usually formed into sharp protrusions with a high aspect ratio. Spindt et al.^2^ used molybdenum to form sharp cones as electron emitters. Others have used different materials, e.g., tungsten^3^. Tungsten wire can be electrochemically etched to form a sharp field emitter^4^. Using silicon microengineering techniques, it is also possible to fabricate sharp silicon structures with good field-emission properties^5–8^.

Recently, carbon nanotubes (CNTs) were investigated as a material for electron field-emission cathodes^9–17^. A CNT is a high aspect-ratio structure. Moreover, CNTs are very good conductors, which makes them ideal candidates for electron field emitters. A single carbon nanotube has been used as a point electron source in high-resolution electron beam instruments^14^. According to the authors, such an electron source is characterized by a stable current and a long lifetime. Furthermore, the energy spread of the emitted electron beam is low. Although CNTs are perfect electron emitters, the fabrication of a single carbon nanotube on a silicon surface is a challenging process. It requires expensive equipment and experience. It is easier to fabricate electron sources using prefabricated CNTs suspended in solution. A CNT layer can be deposited on the surface of a cathode by spray coating^15^, drop drying^16^, or electrophoretic deposition^17^. Cathodes fabricated by these techniques provide a stable electron beam current, but their brightness depends on the shape and size of the deposited films.

**Fig. 1:**
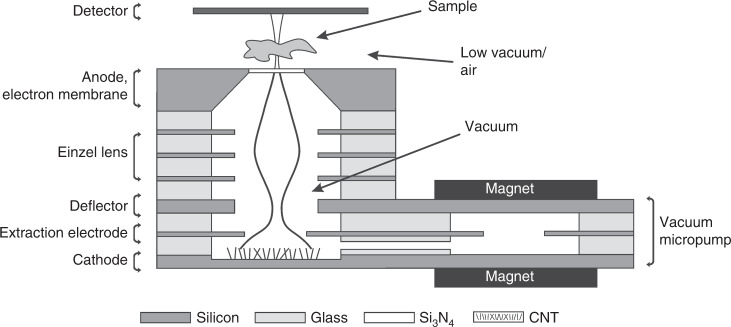
Concept of a miniature MEMS electron microscope. Schematic diagram of the device^18^.

A miniature MEMS electron microscope is being developed at the Wroclaw University of Science and Technology^18^. The concept assumes that all parts of the microscope, the electron gun, electron optics column, and high vacuum micropump, will be fabricated of silicon and glass substrates using MEMS technology (Fig. 1). They will be tightly bonded together in a multilayer anodic bonding process. A high vacuum will be created by an ion-sorption micropump^19^ integrated with the electron-optic microcolumn. The sample and the detector will be placed outside of the high vacuum part of the device. An electron beam with an energy of up to 7 keV will be transmitted to the sample through a 50 nm thick Si_3_N_4_ membrane (anode) that closes the vacuum microchamber. Due to the last factor, i.e., the low energy electron beam and the use of silicon nitride membrane, the electron beam will be scattered, and the final resolution of the developed device will be lower than those of standard, high-resolution TEM or SEM microscopes. However, this device is not designed to compete with highly developed instruments but is a small, mobile microscope with low power consumption. Ongoing research is targeting biological applications such as bacterial identification, cancer cell observation, and research in space.

Since the beginning of the miniature MEMS electron microscope project^18^, electron sources have undergone significant development. The goal was to fabricate a field-emission source on a silicon wafer that can produce a good-quality electron beam (i.e., high and stable e-beam current). We have developed an electrophoretic method of depositing a CNT layer on the surface of a flat silicon cathode^20^. The CNT cathode produced a high electron current, allowing us to conduct the first experiments on electron beam focusing. However, the shape and dimensions of the emission layers were not repeatable. Thus, we started working on improving these parameters. CNTs were still the material of choice, but we obtained some new types of CNT suspensions courtesy of the company OCSiAl, Russia. Tuball Ink™ was particularly interesting due to its ease of transfer and acceptable emission parameters^21^.

Nevertheless, there was a problem with the adhesion of the CNT layer and electrical contact with the silicon surface. We came up with the idea of using an additional adhesive contact layer made by screen printing silver paste^22^. With this technique, we significantly improved the cathode repeatability in terms of shape and dimensions as well as threshold voltage and current stability. These planar CNT cathodes were included in the first MEMS miniature electron microscope model^23^.

However, we were still not fully satisfied, as the CNT emission layers were large (the smallest dimensions: 1 × 1 mm^2^). The electron beam emitted from this cathode was too broad and difficult to focus. In the sample illumination mode of our MEMS microscope, we obtained the first images^23^. Since the images were blurry, we started working on the electron beam scanning system, containing an octupole electrode, in our device. It is known that an electron beam should have a very small spot. Thus, we concluded that the planar CNT cathode had to be replaced with a cathode with a single sharp protrusion.

This article presents the manufacturing process and the results of working parameters in diode and triode configurations of a new type of field-emission electron gun developed for our MEMS electron microscope. Tests of the fabricated MEMS electron microscope demonstrator are also presented.

## Materials and methods

### Fabrication of a sharp silicon protrusion cathode

**Fig. 2:**
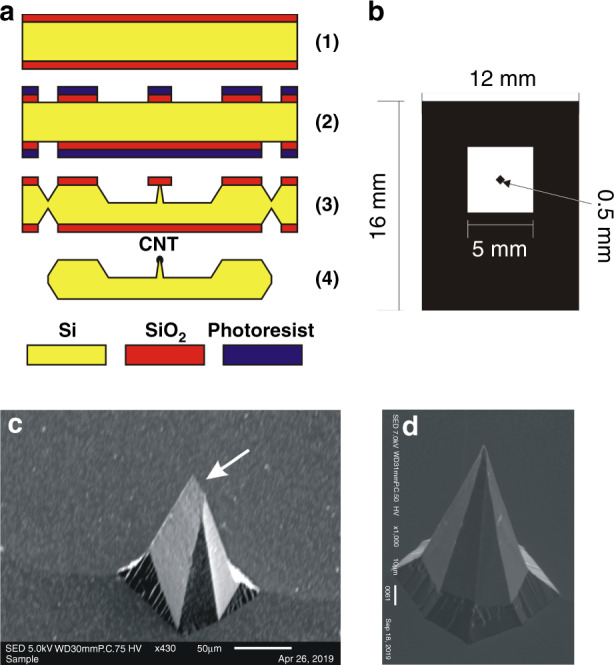
Fabrication of sharp silicon/CNT cathode. **a** Technological steps: (1) oxidation of the silicon wafer, (2) double-sided photolithography, (3) wet anisotropic etching, (4) division of the wafers, removal of the oxide, and deposition of the CNT layer; **b** mask for etching of a single sharp silicon cathode; **c** SEM image of a badly formed tip with an edge (arrow); **d** SEM image of a perfect tip with dimensions <1 µm.

The idea was to make a small protrusion from monocrystalline silicon with the smallest tip diameter possible. The technology used had to be efficient and compatible with other components of the MEMS electron microscope. We used 400 µm thick, n-type, 3″, double-polished silicon wafers with (100) crystallographic orientation. First, the silicon wafer was thermally oxidized (1) (Fig. 2a). A 1-µm-thick SiO_2_ layer was patterned with the double-sided photolithography process (2) and then used as a mask in the anisotropic etching of the silicon. A protrusion and V-grooves to divide the wafer into individual cathodes (3) were fabricated.

To obtain a reasonably high and sharp silicon protrusion in one etching process, etching of the silicon substrate to half its thickness was performed. The 0.5 mm square mask for etching the sharp protrusion was rotated 45° (Fig. 2b) relative to the (100) direction cut of the silicon substrate to expose walls with high crystallographic indexes to build the steep slopes of the structure. There were 16 cathodes (12 × 16 mm^2^) on a single 3-inch wafer. The etching process, performed in an aqueous 10 M KOH solution (80 °C), was controlled, and the sharpness of the tip of the protrusion was checked with an optical microscope.

After a successful process, each sharp silicon protrusion was then characterized by SEM (Jeol JSM IT-100, Japan). The protrusions were approximately the same in shape, i.e., similar to a pyramid with a base edge of ~100 µm, a height of ~200 µm, and tip diameter <5 µm. However, due to the resolution of the mask-making process and the quality of the photolithography, some silicon structures had too much flat surface or had a long edge at the end of the tip (Fig. 2c). The sharpest tips, with radii ranging from 1 to 10 µm, were selected for further experiments (Fig. 2d). Since the electron emission came from a CNT layer deposited on the tip, the difference in tip dimensions was negligible. Moreover, the surface of the silicon protrusion walls was not atomically flat (such as the surface of the polished silicon wafer), which could improve the adhesion and contact between the CNT layer and the silicon tip.

### CNT layer deposition

**Fig. 3:**
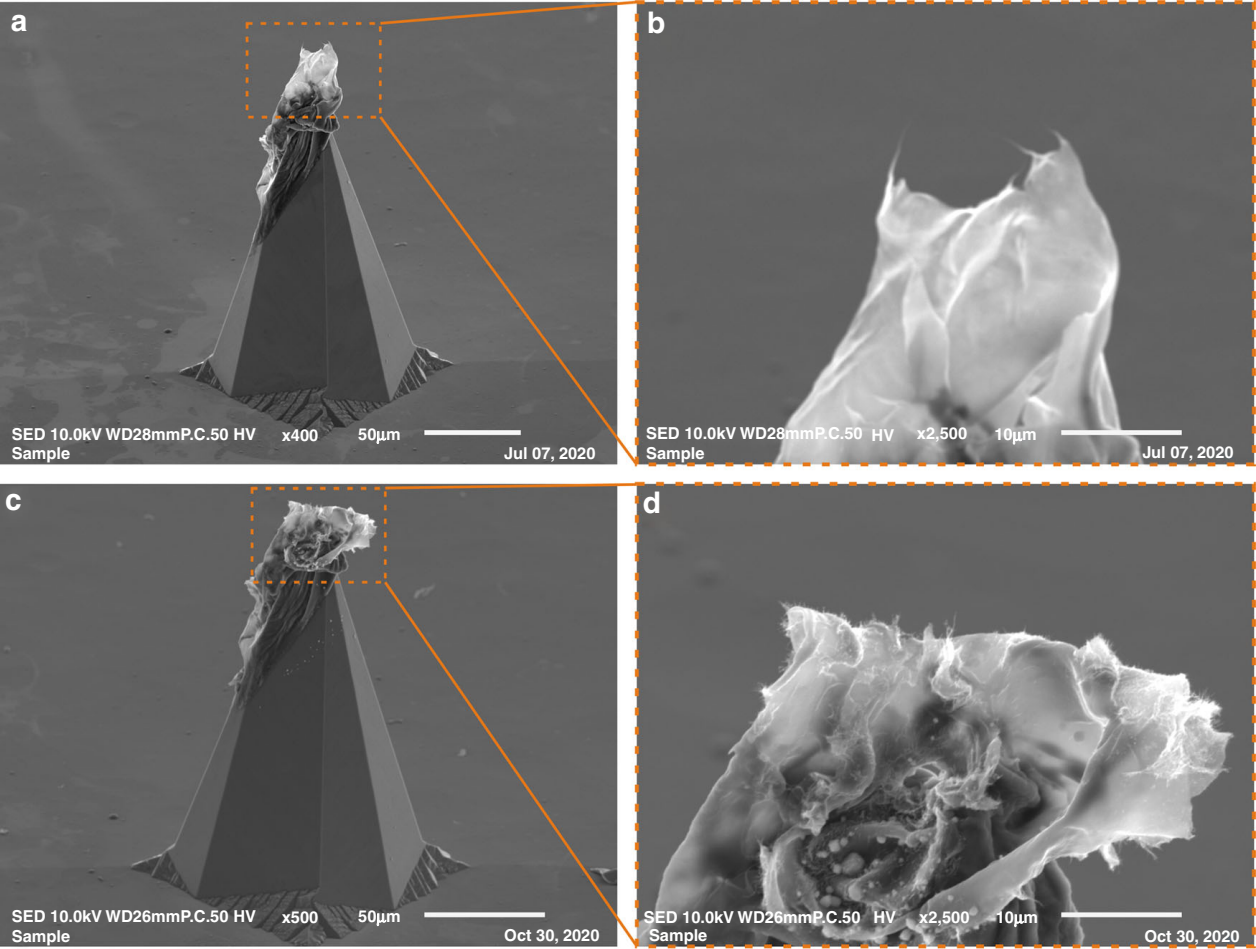
Sharp silicon/CNT cathode used in experiments. **a**, **b** SEM images of the cathode before the experiments: **a** side view of the sharp protrusion of the cathode covered with the CNT layer; **b** magnified image of the end of the cathode; **c**, **d** SEM images of the same cathode after all experiments: (**c**) side view of the silicon protrusion; **d** magnified image of the tip with CNTs protruding from the edges of the layer.

From the several methods we developed, only one fulfilled our requirements, i.e., the CNT thin layer covered only the end of the silicon protrusion in a repeatable way. The method requires heating the silicon chip (220 °C) before CNT ink deposition. The CNT ink was a 0.2 wt% suspension of SWNTs (single-walled carbon nanotubes) in deionized water (DI) with a 2% addition of surfactant. The end of a nylon thread (100 µm diam.) dipped in the CNT suspension was used to place the smallest possible drop of CNT suspension onto the end of the tip. We used nylon thread because it bent when it touched the silicon protrusion. Using a precision pipette or metal wire could break the tip of the protrusion. As the CNT suspension had been deposited, rapid water evaporation limited the reflow of the droplet over the entire silicon surface. Moreover, evaporation of the water made the CNT layer more porous and rugged, and the CNT nanotubes could be positioned closer to vertical. The method was repeatable and allowed the deposition of the CNT layer only at the end of the tip (Fig. 3a), which was the goal. However, the appearance of the CNT layer as observed by SEM was slightly different for the successively produced sharp silicon/CNT cathodes. Nonetheless, the performance of the cathodes was repeatable. Moreover, each CNT layer contained tangled carbon nanotubes protruding from the edges (Fig. 3b), which could explain the very good emission properties of such fabricated cathodes. The silicon protrusion limited the dimensions of the emission area to a few micrometers, and the emission mainly came from CNTs aligned vertically on this area.

After all measurements, we examined the sharp silicon/CNT cathode again using SEM. The changes in its tip were visible; the CNT layer looked like it had “exploded” (Fig. 3c). It changed in shape, but single strands of CNTs still protruded from the edges of the film (Fig. 3d). This is a good result that suggests a long lifetime for the cathodes made with the developed technology.

## Results and discussion

### Diode configuration

**Fig. 4:**
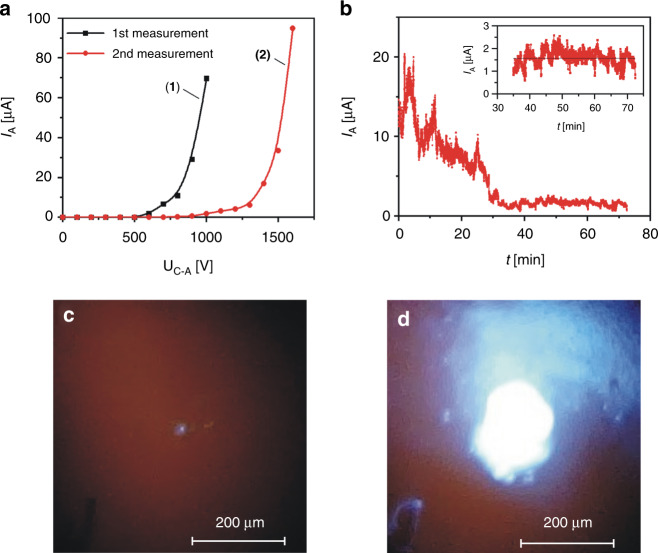
Results of measurements in a diode configuration. **a** Anode current as a function of the cathode–anode voltage: (1) the first measurement; (2) the second measurement after over an hour of continuous work of the cathode; **b** the anode current as a function of time for the sharp silicon/CNT cathode; *U*_C-A_ = 900 V; **c**, **d** electron beam spot observed on phosphor screen: **c** the spot at *U*_C-A_ = 1100 V, **d** the spot at *U*_C-A_ = 1500 V.

First, we measured the electron emission from the newly developed sharp silicon/CNT cathode in a diode configuration. The anode was made of glass with the ITO layer covered with a phosphor (ZnS:Ag). A 1.1 mm thick borosilicate glass spacer with a 5 × 5 mm^2^ hole in the center was used to separate the electrodes. With this configuration (Supplementary Fig. S1), we could measure the electron beam current and observe the electron beam cathodoluminescence. The test structure was put inside a high vacuum chamber, which was then evacuated to 1 × 10^−5^ mbar. A high negative voltage up to 1600 V was applied at the cathode. The anode was at the ground state. The anode current was measured with increasing cathode–anode voltage (every 100 V). The first measurement was performed ~30 min after the deposition of the CNT layer on the cathode. Measurements of the anode current (*I*_A_) were performed five times for each set voltage (*U*_C-A_) to average the current value and decrease the measurement error. Most of the fabricated field cathodes had an initial threshold voltage below 1000 V. In the example plot (Fig. 4a, 1), in the first measurement, the cathode threshold voltage was 500 V. The anode current was high, reaching 70 µA at *U*_C-A_ = 1000 V.

After the first measurement, the cathode–anode voltage was set at 900 V, setting a moderate anode current in this configuration. The anode current was then measured for 72 min, and the stability of the current over time was observed. The results show that the current decreases for the first 30 min (Fig. 4b). High fluctuations in the anode current value (a phenomenon typical of field emission) were visible, and over time, the fluctuations diminished, reaching a plateau after 30 min of continuous work. Then, the current was stable with an average value of *I*_A_ = 1.6 µA, but it still fluctuated over this value (Fig. 4b, inset). The initial decrease in the anode current value was a result of ion bombardment of the CNTs protruding from the tip. At first, the number of vertically arranged CNTs was high, resulting in a high current. The ions generated in the residual gas damaged the CNT layer, which lowered the current value. After a certain time, steady state was achieved between the destroyed and newly disclosed nanotubes in the layer, and a stable current was observed (Fig. 4b, inset). The process of the current value decreasing in these cathodes was faster than that in the case of the previous flat cathodes^22^. It took ~30 min to obtain a more stable value. Previously, it took several hours.

To see how the process of 72 min of continuous operation affected the cathode, a second measurement of the emission characteristics was performed. The most significant change was observed in the threshold voltage, which rose to 900 V. The anode current was still high (*I*_A_ = 95 µA at *U*_C-A_ = 1600 V), whereas the rest of the plot looked similar in shape (Fig. 4a, 2).

After the current-voltage measurements, we started to observe the electron beam spot at the phosphor screen of the same cathode. Through the glass anode, we could see a sharp silicon/CNT cathode. After a voltage was applied to the cathode, the effects of electron emission from the tip could be seen (Fig. 4c, d). The blue light generated on the phosphor layer was directly over the tip. It is best to keep the anode current and cathode–anode voltage low (*U*_C-A_ = 1100 V, *I*_A_ = 3 µA) to obtain a small electron beam spot (Fig. 4c). Under these conditions, the spot was approximately the same dimension as the emission tip (~10 µm). When the cathode–anode voltage was increased to 1500 V and the anode current reached 33 µA, the observed spot size was much larger (Fig. 4d). This was probably due to the emission of electrons from the sides of the tip under the influence of a stronger electric field.

### Triode configuration

Next, we tested the same sharp/CNT cathode in a triode configuration. We added a third silicon extraction electrode (gate) with a square central opening of 2 × 2 mm^2^, fabricated using the same technology, and a second glass spacer. In the triode configuration, the cathode voltage determines the electron beam energy, and the gate voltage controls the value of the beam current reaching the anode. First, we performed measurements of the gate controlling operation. The anode was set at the ground state, and the cathode was supplied with negative voltage. Three different cathode voltages were chosen (−1000 V, −1600 V, and −1900 V) for measurements defining the electron beam energy. In each measurement, only the gate voltage was changed, and the cathode current (*I*_C_) was recorded. First, the gate voltage was set at the same value as the cathode (*U*_C-G_ = 0 V), and then it was decreased every 100 V (increasing the cathode-gate voltage difference – *U*_C-G_) (Supplementary Fig. S2).

**Fig. 5:**
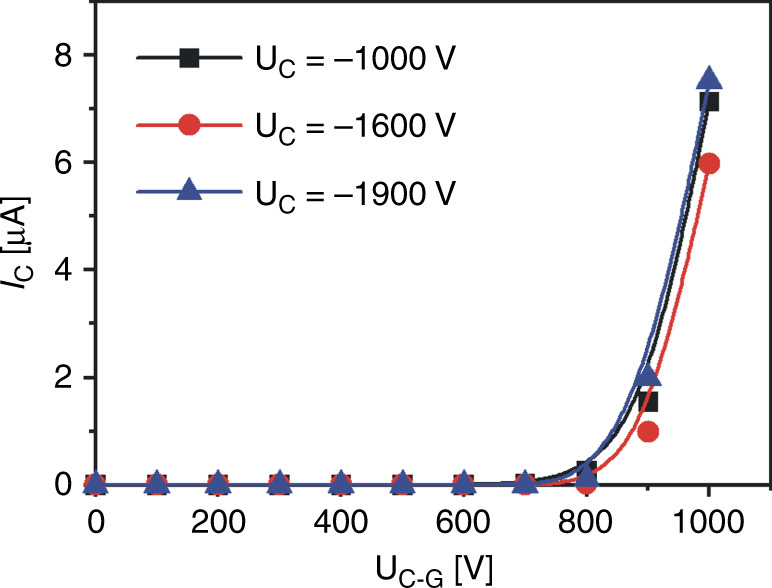
Results of measurements in a triode configuration. The cathode current as a function of the cathode-gate voltage *U*_C-G_ for 3 cathode voltages.

For each cathode voltage, the emission started when *U*_C-G_ = 700 V, yielding similar cathode current values (e.g., at *U*_C-G_ = 900 V: for *U*_C_ = −1000 V, *I*_C_ = 1.5 µA; for *U*_C_ = −1600 V, *I*_C_ = 1 µA; for *U*_C_ = −1900 V, *I*_C_ = 2 µA) (Fig. 5). This confirmed the controlling role of the gate electrode in electron beam limitation. Moreover, using these values, we could estimate the minimal lifetime of such a sharp silicon/CNT cathode. In the MEMS electron microscope, we wanted to work with an e-beam current of 1 µA. This current value should give us a good electron beam signal after passing through the thin (50 nm) silicon nitride membrane. Such a value can be achieved at *U*_C-G_ = 900 V.

Considering that we want to use a 4 keV beam, we need to supply the cathode with U_C_ = −4000 V and the gate with *U*_G_ = −3100 V. From the diode measurements, we know that the anode current reaches the stable plateau after 30 min of continuous work and is stable for at least 40 min. Therefore, with the mentioned voltages, the electron gun should supply the microscope at 1 µA for 40 min. We can assume that after that time, the current value drops, and to keep its value at the desired level, we need to reduce the gate voltage by 100 V. This operation gives another 40 min of stable current. We can repeat this until the gate voltage reaches 0 V. The total amount of time of stable work for an e-beam energy of 4 keV and 1 µA current is 1280 min (over 21 h), which is an excellent result.

### Stabilization of the e-beam current

Changing the gate electrode voltage makes it possible to reduce the electron beam current and extend the lifetime of the electron gun. Moreover, we can stabilize the anode current by controlling the gate voltage in the feedback loop with the anode current value. For the experiment, we used the cathode in a triode configuration from previous measurements but changed the ITO glass anode to a silicon anode (Supplementary Fig. S3).

**Fig. 6:**
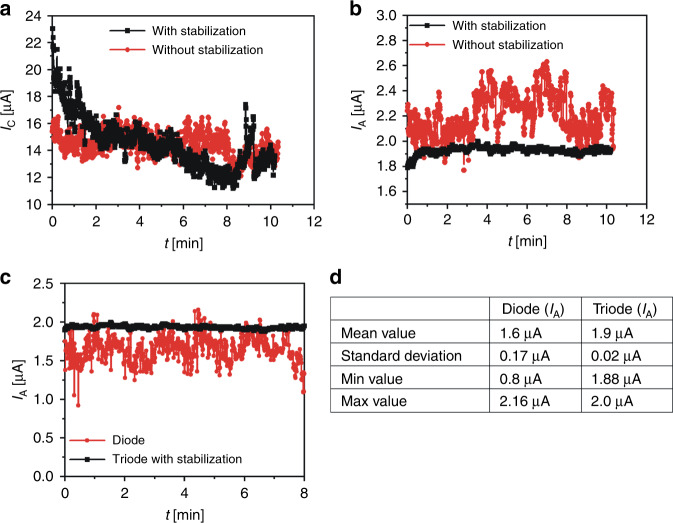
Results of experiments with electron beam current stabilization. **a**, **b** Test of the sharp silicon/CNT cathode in the triode configuration using a circuit with and without a stabilization feedback loop: **a** cathode current vs. time; **b** anode current vs. time; **c** comparison of the anode current vs. time in a diode (red plot) and triode (black plot) configurations; **d** electron beam current for both configurations.

First, anode current measurements were performed with a custom-made voltage supply. The voltage supply is made of 2 miniature DC/HVDC converters (XP Power, UK). The converters give high positive voltage output up to 3 kV. The first converter supplies the anode and the second gate electrode. The cathode remains at the ground state (Supplementary Fig. S3a). The voltage supply allows the anode current to be monitored and in the feedback loop, using an analog circuit, regulates the gate voltage to maintain the anode current stability. In the measurements, the anode voltage was set at 1.7 kV (i.e., the electron beam energy was set at 1.7 keV), and the anode current value was set at 1.9 µA. The gate voltage was set by the second converter to maintain the stable anode current. The cathode and anode currents were measured for over 10 min (Fig. 6, black plot).

Second, the measurements were repeated using laboratory high voltage supplies without a feedback loop (Supplementary Fig. S3b). The anode voltage was set at 1.7 kV, and the gate was supplied with a voltage that allowed it to reach the same anode current level as previously described (*U*_G_ = 2200 V). Again, the cathode and anode currents were measured for over 10 min (Fig. 6, red plot).

The cathode currents in both cases were not stable (Fig. 6a), but the average value was almost the same at ~14.50 µA (14.53 µA for a beam with a stabilization feedback loop and 14.47 µA for a beam without stabilization). However, when we look at the anode current plot, we can see the difference (Fig. 6b). The gate voltage adjustment by a voltage supply with a feedback loop maintained the anode current at 1.9 µA. A small increase in the anode current was visible at the beginning of the plot, and stabilization took ~1 min to start. For measurements without stabilization, the fluctuations in the anode current were high. The anode current was well over 1.9 µA and reached a value of 2.6 µA.

The controlling role of the gate electrode can also be observed by comparing (Fig. 6c) the part of the anode current in the stable region (the chosen 8 min from the inset of Fig. 4b) measured in the diode configuration and part of the anode current in the stabilizing feedback loop (the last 8 min from Fig. 6b) measured in the triode configuration. The average value of the anode current measured in the diode configuration was 1.6 µA. Its standard deviation was 0.17 µA, which means that the current fluctuation of the electron beam in the stable plateau reached 10%. Looking at similar values for the triode configuration (average current of 1.9 µA with a standard deviation of 0.02 µA), it can be noticed that using the gate electrode and applying a feedback loop could reduce the e-beam current fluctuations to ~1% (Fig. 6d). This is an excellent result in terms of using a developed electron gun for MEMS electron microscopy.

### Testing of the MEMS electron source demonstrator

**Fig. 7:**
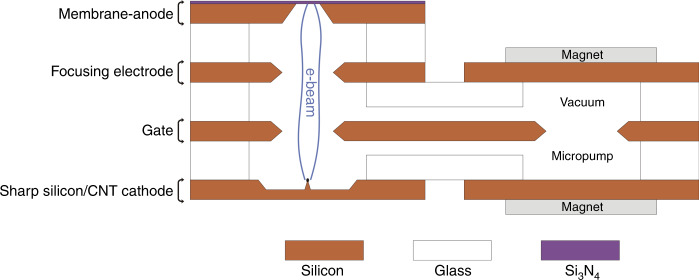
Concept of a MEMS electron source. Schematic cross-section of the device.

The final aim of the newly developed electron gun is its use in a working MEMS electron microscope demonstrator. For this experiment, the structure of the MEMS electron microscope was limited compared to the main concept (Fig. 1). The demonstrator, in the form of an electron source (Fig. 7), contains only 4 electrodes in the electron optics column: a sharp silicon/CNT cathode, a gate, and a focusing electrode, both with 2 × 2 mm^2^ holes, and an anode with a very thin (50 nm) silicon nitride membrane (250 × 250 µm^2^). The positions of the holes and the membrane are aligned with the position of the cathode tip. The high vacuum ion-sorption micropump contains 3 electrodes: 2 square cathodes and an anode, which is fabricated on the same silicon chip as the gate electrode of the microcolumn. The anode has a 4 × 4 mm^2^ hole centered with respect to the cathodes. The silicon electrodes were fabricated using microsystem technology, and the detailed procedure can be found in our previous publications^20,24^. The silicon electrodes are separated from each other by borosilicate glass spacers with 5 × 5 mm^2^ openings.

**Fig. 8:**
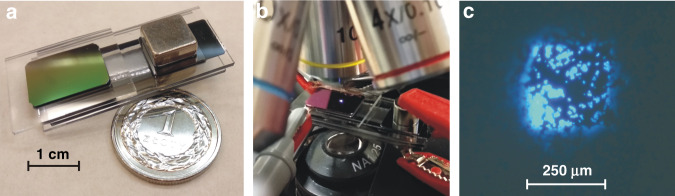
Demonstrator of the MEMS electron source. **a** Photo of the demonstrator integrated with an ion-sorption micropump; **b** demonstrator under an optical microscope (blue light is visible in the center of the anode); **c** cathodoluminescence of the ZnS:Ag phosphor deposited on the silicon nitride membrane (image of an optical microscope).

The silicon and glass parts were joined together using a multilayer anodic bonding process (process conditions: 450 °C, 1500 kV). The last anodic bonding was conducted in a vacuum because the structure needs to have an initial vacuum in the range of 10^−1^ mbar to start a high vacuum micropump operation. The fabricated demonstrator (Fig. 8a) was connected to a voltage supply. First, we turned on the micropump. We observed the ion current to drop down to 1 µA, indicating that a high vacuum (10^−5^ mbar) was generated inside the miniature device. After 5 min of micropump operation, the voltages on individual electrodes (cathode, gate, focusing, and anode) were applied as *U*_C_ = −3 kV, *U*_G_ = −2 kV, *U*_F_ = −3 kV, *U*_*A*_ = 0 V. We measured the cathode current of 2 µA, which confirmed that the developed sharp silicon/CNT cathode worked properly in the fabricated high vacuum self-sufficient device.

To test the performance of the MEMS electron source, a thin layer of ZnS:Ag phosphor was placed on the membrane. The electron beam generated by the cathode was transmitted through a thin membrane, and the cathodoluminescence of the phosphor was observed under an optical microscope (Fig. 8b, c). This experiment confirmed that a miniature electron beam device could be fabricated and operated under atmospheric pressure without external high vacuum chambers (Fig. 8b).

## Conclusions

We achieved a significant step in designing and developing a miniature MEMS electron microscope. We fabricated and tested a newly developed electron-optical microcolumn with a sharp silicon/CNT field-emission cathode. Its fabrication process follows the technology of miniature device under development, built from silicon electrodes and glass spacers. The electron gun contains a silicon cathode with a single very sharp protrusion with a bundle of disordered CNTs deposited on its end. The fabrication technology of the sharp protrusion and the deposition process of the CNT layer are repeatable, producing cathodes with similar dimensions and electron emission areas of less than 10 µm. Moreover, the electrical properties of such fabricated cathodes are very good, i.e., with a low threshold voltage (<1000 V) and a high electron beam current. In comparison, other types of field emitters, such as tungsten or molybdenum tips or single carbon nanotubes, are characterized by a lower threshold voltage of ~100 V, but similar electron beam current values are obtained. The main difference in the design of the developed cathode lies mainly in the need to use materials compatible with MEMS technology. Another difference concerns the arrangement of the MEMS electron gun elements. Typically, the sharp tip of the cathode is very close to the extraction electrode, but the developed silicon-glass design uses a thicker glass spacer, which increases the value of the threshold voltage.

Experiments carried out for a sharp silicon cathode/CNT in a diode configuration showed that the emission of electrons from the CNT layer stabilizes quickly and that after 30 min of continuous operation, the electron beam current becomes stable, with fluctuations of ~10%. This stability was exhibited for more than 40 min of measurement. Continuous cathode operation had little effect on the cathode performance; the threshold voltage was increased from 500 to 900 V, but the measured e-beam current was still high. Furthermore, the very good brightness of the electron beam current was confirmed. The electron beam spot observed at the ZnS:Ag phosphor screen had a diameter close to the emission area (<10 µm) when the current was a few µA.

The triode configuration experiments confirmed the significant role of the extraction (gate) electrode. Using the gate electrode, it was possible to set the beam energy and limit the electron beam current. Moreover, the lifetime of the cathode for the required energy could be increased by changing the gate voltage. Rough calculations for the 4 keV beam energy showed that a stable electron beam current of 1 µA could be emitted from the CNT cathode for 1280 min (over 21 h), which is an excellent result.

The gate electrode could be used for the stabilization of the electron beam. Using a custom-made voltage supply that changed the gate voltage in the feedback loop in relation to the anode current, the electron beam fluctuations could be limited to 1%. This value of electron beam current fluctuation is acceptable for SEM use.

Imaging of the sharp silicon cathode/CNT with SEM after all the tests (a long work time) showed changes from the original structure. The CNT layer was heavily damaged (by ion bombardment of residual gases, the thermal effect of the flowing current); however, some strands of CNTs protruded from the edges of the layer. This showed that the deposited CNT layer was dense and resistant to ion bombardment, confirming the cathode lifetime estimation.

The most important experiment presented was the fabrication of the MEMS electron source demonstrator, which included a newly developed sharp silicon/CNT cathode that worked in air. The device was made according to assumptions about silicon electrodes and glass spacers using MEMS technology. The results showed that the developed cathode withstood the anodic bonding process and worked well in the fabricated structure. The measured current for the field-emission cathode at 3 kV was 2 µA. Moreover, we observed the cathodoluminescence of the ZnS:Ag phosphor deposited on the outer surface of the membrane, which confirmed that the electron beam was transmitted from the microcolumn through the nitride membrane to the sample. In this form, the miniature MEMS electron source can be used for electron irradiation of samples, for cathodoluminescence study of different materials, and as an electron ionization tool for miniature mass spectrometers.

Having developed an electron gun with satisfactory parameters that works in a closed high vacuum microcolumn, we are one step closer to fabricating a final MEMS electron microscope. The electron beam scanning system for this miniature device is currently underway.

## Supplementary information

Supplementory material - measurements schematics

## References

[1] Chang THP (1996). Electron beam technology – SEM to microcolumn. Microelectron. Eng..

[2] Spindt CA, Brodie I, Humphrey L, Westerberg ER (1976). Physical properties of thin-film field emission cathodes with molybdenum cones. J. Appl. Phys..

[3] Park YJ (2000). Fabrication of Spindt-type tungsten microtip field emitter arrays with optimized aluminum parting layers. J. Vac. Sci. Technol. B.

[4] Chang W-T (2012). Method of electrochemical etching of tungsten tips with controllable profiles. Rev. Sci. Instrum..

[5] Marcus RB (1990). Formation of silicon tips with <1 nm radius. Appl. Phys. Lett..

[6] Ravi TS, Marcus RB, Liu D (1991). Oxidation sharpening of silicon tips. J. Vac. Sci. Technol. B.

[7] Boswell EC, Huq SE, Huang M, Prewett PD, Wilshaw PR (1996). Polycrystalline silicon field emitters. J. Vac. Sci. Technol. B.

[8] Dey RK, Shen J, Cui B (2017). Oxidation sharpening of silicon tips in the atmospheric environment. J. Vac. Sci. Technol. B.

[9] Bocharov GS, Eletskii AV (2013). Theory of carbon nanotube (CNT)-based electron field emitters. Nanomaterials.

[10] Cheng Y, Zhou O (2003). Electron field emission from carbon nanotubes. C. R. Phys..

[11] Leberl D (2013). Characterization of carbon nanotube field emitters in pulsed operation mode. J. Vac. Sci. Technol. B.

[12] Eletskii AV (2010). Carbon nanotube-based electron field emitters. Phys. Uspekhi.

[13] Bonard JM (2002). Carbon nanotube films as electron field emitters. Carbon.

[14] de, Jonge N, Lamy Y, Schoots K, Oosterkamp TH (2002). High brightness electron beam from a multi-walled carbon nanotube. Nature.

[15] Hwang JW, Mo CB, Jeong YJ, Hong SH (2011). Fabrication and characterization of a 3D-Structured field emitter using carbon nanotube. J. Nanosci. Nanotechnol..

[16] Heo SH, Ihsan A, Yoo SH, Ali G, Cho SO (2010). Stable field emitters for a miniature X-ray tube using carbon nanotube drop drying on a flat metal tip. Nanoscale Res. Lett..

[17] Boccaccini AR (2006). Electrophoretic deposition of carbon nanotubes. Carbon.

[18] Krysztof, M., Grzebyk, T. P., Górecka-Drzazga, A., Adamski, K. & Dziuban, J. A concept of fully integrated MEMS-type electron microscope. In *27th International Vacuum Nanoelectronics Conference (IVNC): Technical Digest, IEEE, 6-10 July 2014, Engelberg, Switzerland* (2014).

[19] Grzebyk T, Górecka-Drzazga A, Dziuban JA (2014). Glow-discharge ion-sorption micropump for vacuum MEMS. Sens. Actuators A.

[20] Krysztof M, Grzebyk TP, Górecka-Drzazga A, Adamski K, Dziuban J (2018). Electron optics column for a new MEMS-type transmission electron microscope. Bulletin of the Polish Academy of Sciences. Tech. Sci..

[21] Laszczyk, K. et al. From a microtip to a planar cathode – an electron source with a simplified technology. In *15th International Conference on Optical Sensors and Electronic Sensors, COE 2018, 17–20 June 2018, Warsaw, Poland, IEEE* cop*.* 1–4 (2018).

[22] Laszczyk, K., Krysztof, M., Dziuban, J., & Górecka-Drzazga, A. The stable and long lifetime planar field emission cathode made of a CNT ink. In *31st International Vacuum Nanoelectronics Conference, IVNC 2018: Technical Digest, Kyoto, Japan 9–13 July 2018/Japan Society for the Promotion of Science, IEEE* cop. 1–2 (2018).

[23] Krysztof M, Grzebyk TP, Górecka-Drzazga A (2020). Preliminary research on imaging in MEMS electron microscope. Meas. Sci. Technol..

[24] Krysztof M (2018). Technology and parameters of thin membrane-anode for MEMS transmission electron microscope. J. Vac. Sci. Technol. B.

