# Supplementary material for: Field-emission electron gun for a MEMS electron microscope
Source: Microsyst Nanoeng. 2021 Jun 1;7:43. doi: 10.1038/s41378-021-00268-9 (PMC8433145; doi:10.1038/s41378-021-00268-9)
Supplement: Supplementary file 1 — Supplementory material - measurements schematics [file 41378_2021_268_MOESM1_ESM.docx]

SUPPLEMENTARY INFORMATION: FIELD EMISSION ELECTRON GUN FOR MEMS ELECTRON MICROSCOPE

Michał Krysztof*
Wroclaw University of Science and Technology,
Faculty of Microsystem Electronics and Photonics,
Department of Microsystems
ul. Z. Janiszewskiego 11/17, 50-372 Wrocław, Poland
*corresponding author e-mail: michal.krysztof@pwr.edu.pl


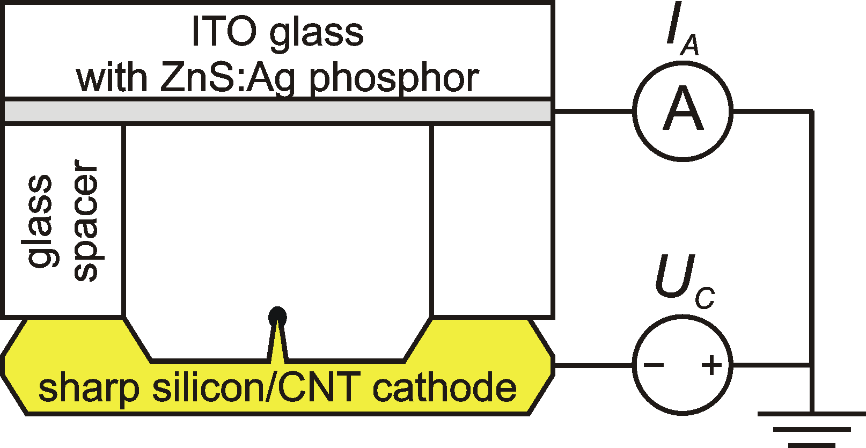


***Fig. S1. Measurement schematics in diode configuration experiments.***


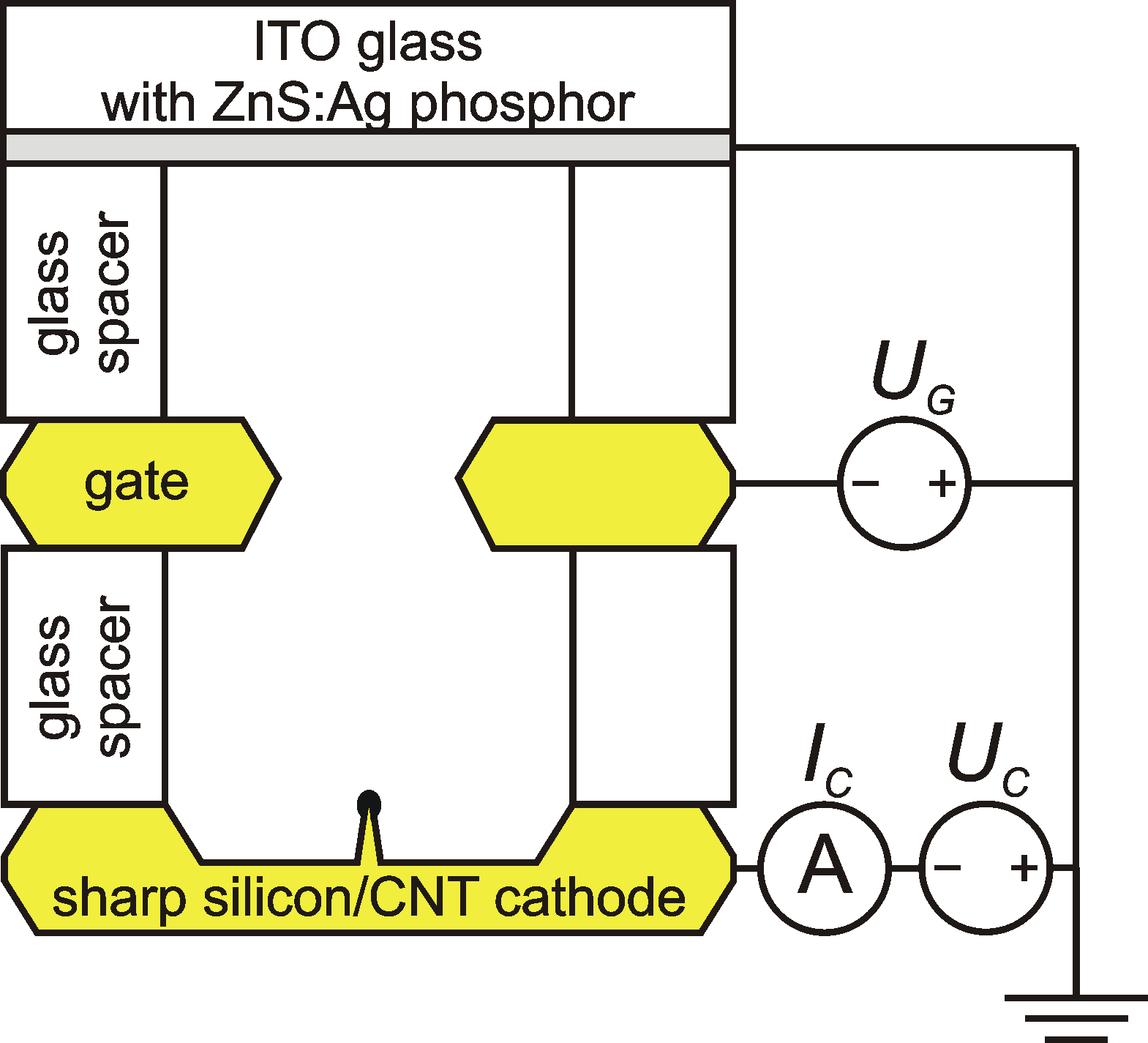


***Fig. S2. Measurement schematics in triode configuration experiments.***

~~
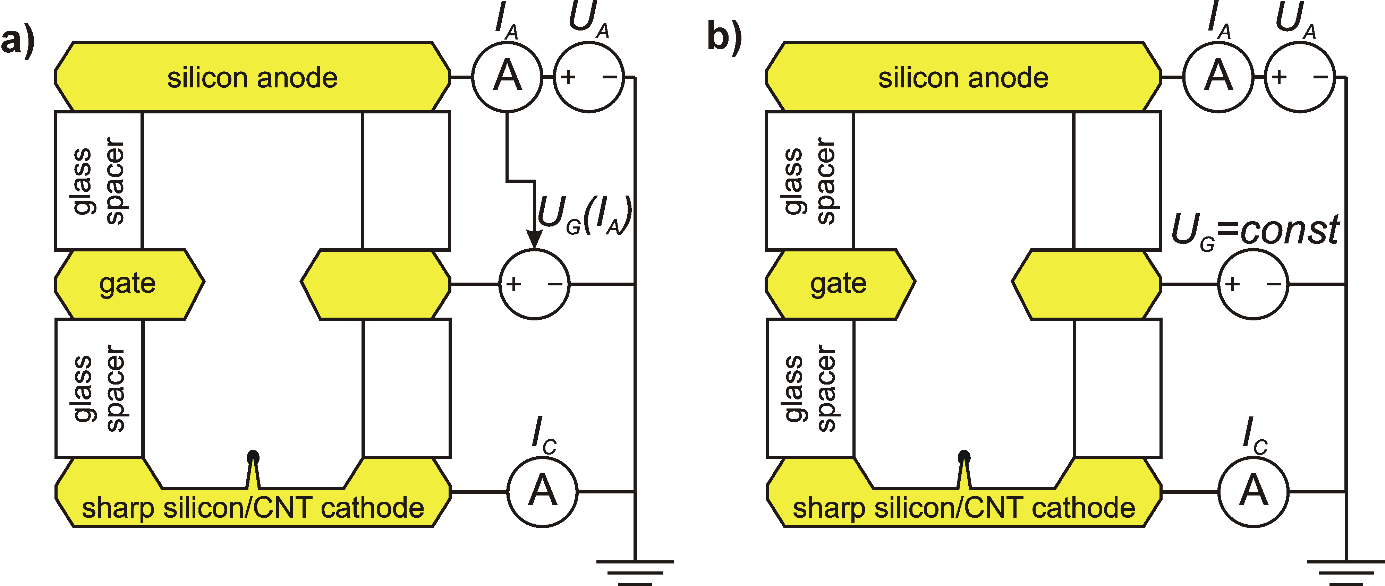
~~

***Fig. S3. Measurements schematics in triode configuration experiment of the anode current stabilization: a) the first experiment with the use of a stabilization feedback loop, b) the second experiment with the use of laboratory high voltage supplies.***
